# Supplementary material for: MTA, an RNA m6A Methyltransferase, Enhances Drought Tolerance by Regulating the Development of Trichomes and Roots in Poplar
Source: Int J Mol Sci. 2020 Apr 2;21(7):2462. doi: 10.3390/ijms21072462 (PMC7177244; doi:10.3390/ijms21072462)
Supplement: Supplementary file 1 [file ijms-21-02462-s001.pdf]

Supplementary Materials

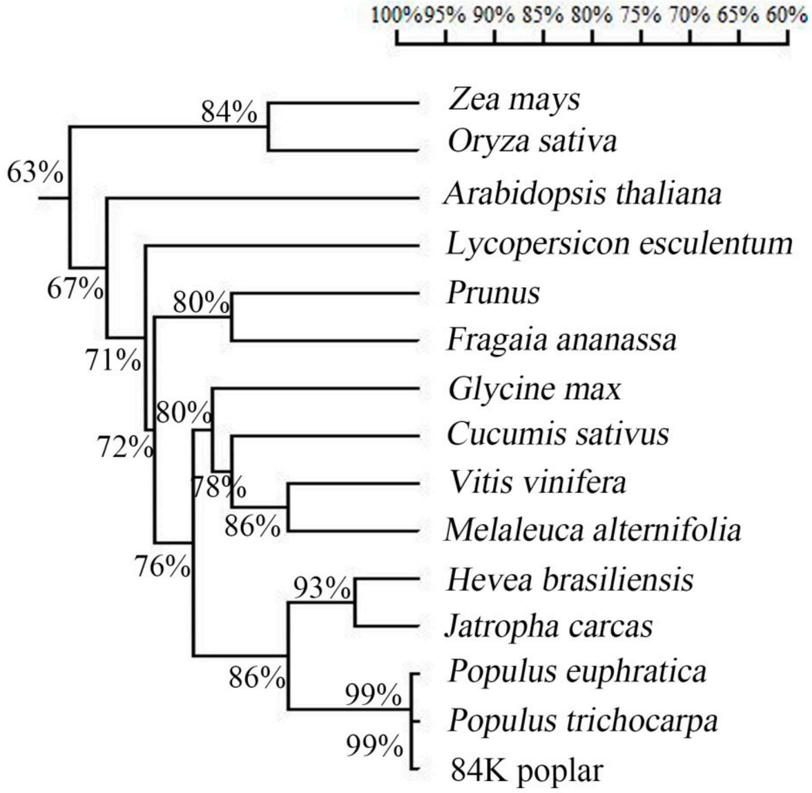

Figure S1. Phylogenetic tree of different MTA protein family members and amino acid sequence alignment of PtrMTA. Phylogenetic analysis of the PtrMTA homologs from *Zea mays*, *Oryza sativa*, *Arabidopsis thaliana*, *Lycopersicon esculentum*, Prunus persica, *Fragaria ananassa*, *Glycine max*, *Vitis vinifera*, *Melaleuca alternifolia*, *Hevea brasiliensis*, *Jatropha carcass*, *Populus euphratica*, *Populus trichocarpa* and 84K poplar.

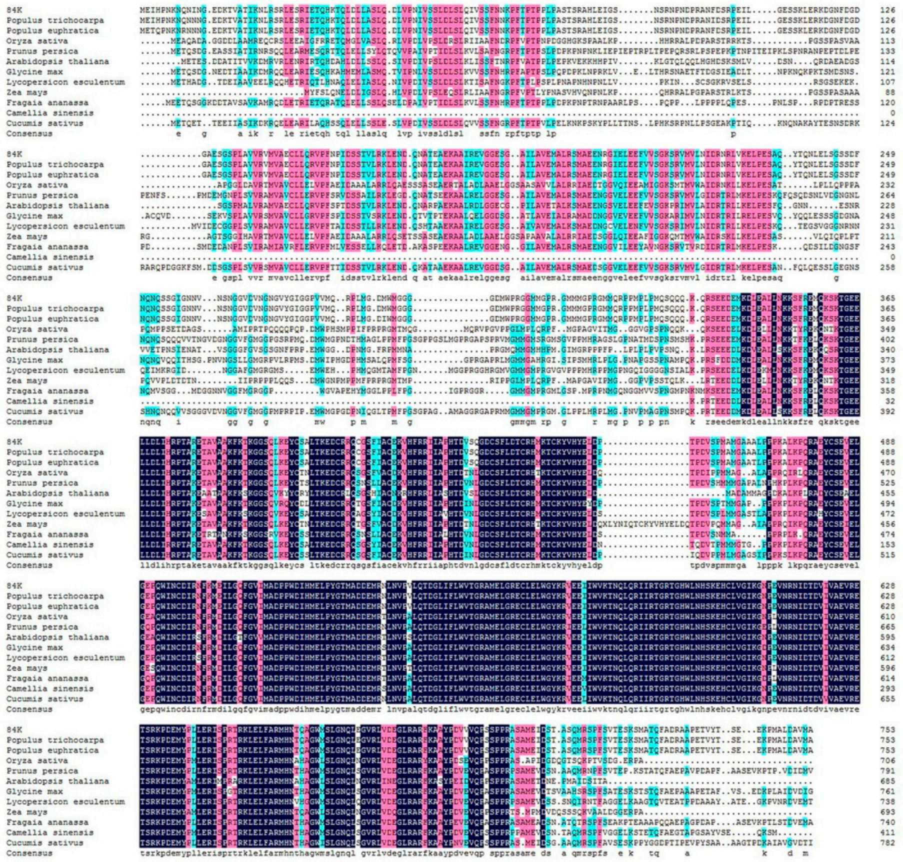

Figure S2. MTA was highly conserved among various plant species. Alignment of amino acid sequences of PtrMTA and its orthologs from various plant species. Sequences of MTA orthologs from 84K poplar, *Populus trichocarpa*, *Populus euphratica*, *Oryza sativa*, *Prunus persica*, *Glycine max*, *Lycopersicon esculentum*, *Fragaria ananassa*, *Arabidopsis*, *Zea mays*, *Camellia sinensis* and *Cucumis sativus* were obtained from the NCBI and Phytozome databases. Conserved residues are shown in black.

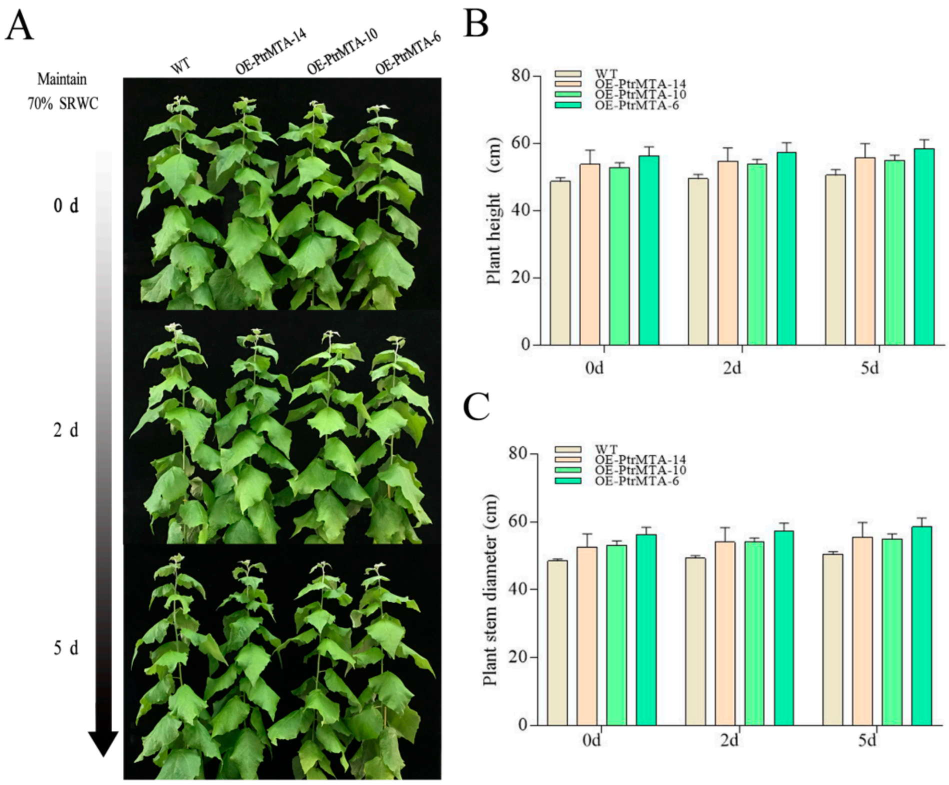

Figure S3. Growth status of WT and *PtrMTA*-overexpressing plants under normal conditions. (A) Phenotypic analysis under normal conditions. (B) Plant height statistics of wild type (WT) and *PtrMTA*-overexpressing plants under normal conditions. (C) Plant stem diameter statistics of wild type (WT) and *PtrMTA*-overexpressing plants under normal conditions. Error bars are means ± SE (n = 4). All asterisks denote significant differences: $^{**}p < 0.01$.

Table S1. Statistics on the number of trichomes in the 20 areas (1500 μm * 1000 μm) of leaves in WT and transgenic plants.

Sample	Statistical data	Average
WT	08.08.10.05.11.08 07.09.10.10.05.07.07.09.10.08.12.14.10.14	9
PtrMTA-14	12.14.11.14.14.14.11.10.10.14.16.15.13.12.13.15.12.12.11.10	13
PtrMTA-10	15.16.18.12.13.14.16.15.15.16.18.12.14.19.20.17.10.12.14.16	14
PtrMTA-6	28.30.24.20.23.20.24.25.31.18.20.18.16.21.23.19.16.32.28.25	23

Table S2. Analysis of the *PtrMTA* promoter functional components.

Sequence	Organism	Element number	Position from ATG	Function
ACGTG	*Arabidopsis thaliana*	2	−1377, −1396	cis-acting element involved in the abscisic acid responsiveness
ATAGAAATCAA	*Glycine max*	1	−87	binding site of AT-rich DNA binding protein (ATBP-1)
AACGAC	*Brassica oleracea*	1	−532	auxin-responsive element
CCATCTTTTT	*Nicotiana tabacum*	2	−433, −2600	cis-acting element involved in salicylic acid responsiveness
GGTTAA	*Arabidopsis thaliana*	4	−45, −201, −1809, −1966	light responsive element
CAACTG	*Arabidopsis thaliana*	1	−2641	MYB binding site involved in drought-inducibility
AACCTAA	*Petroselinum crispum*	3	−354, −400, −2544	MYB binding site involved in light responsiveness
ATTAAT	*Petroselinum crispum*	3	−458, −1923, −2066	part of a conserved DNA module involved in light responsiveness
GTTTTCTTAC	*Nicotiana tabacum*	1	−1220	cis-acting element involved in defense and stress responsiveness
CCCCCG	*Zea mays*	1	−2365	enhancer-like element involved in anoxic specific inducibility
TACGTG	*Arabidopsis thaliana*	1	−1376	cis-acting regulatory element involved in light responsiveness

Table S3. Primers used for PCR analysis.

Primer name	Primer sequence
F-cloning PtrMTA	5′ GGGGTACCATGGAGATTCATCCAAACAAGAAC 3′
R-cloning PtrMTA	5′ CGGGATCCTCAGCCAGCCATAACAGCATCTAA 3′
F-qRT-PtrMTA	5′ TGCTATGGAGCTTGGACGTG 3′
R-qRT-PtrMTA	5′ AGGGTACATCTCGTCTGGCT 3′
F-18S	5′ CGAAGACGATCAGATACCGTC 3′
R-18S	5′ TTTCTCATAAGGTGCTGGCGG 3′
F-PtrMTA-GFP	5′ GGGGTACCATGGAGATTCATCCAAACAAGAAC 3′
R-PtrMTA-GFP	5′ CGGGATCCGCCAGCCATAACAGCATCTAA 3′
F-PtrFIP37-mCherry	5′ CGGGGTACCATGGCATCGCACAACCATCT 3′
R-PtrFIP37-mCherry	5′ GCTCTAGAGTTGACCTCAGCTTCCTTC 3′
F-identification PtrMTA	5′ AATTGAGACTTTTCAACAAAGGG 3′
R-identification PtrMTA	5′ TCAAAGCCATTTCCACCG 3′

Table S4. GenBank IDs of the PtrMTA homologues proteins

Species	GenBank IDs
Populus trichocarpa	7477828
Zea mays	100281493
Oryza sativa	4330284
Arabidopsis thaliana	826670
Lycopersicon esculentum	543972
Prunus persica	110758914
Fragaria ananassa	101294523
Glycine max	100788519
Vitis vinifera	100256414
Melaleuca alternifolia	114279743
Hevea brasiliensis	100669519
Jatropha carcass	105645610
Populus euphratica	105127270
